# Mucinous cystadenoma and carcinoid tumor arising from an ovarian mature cystic teratoma in a 60 year-old patient: a case report

**DOI:** 10.1186/s13256-024-04603-2

**Published:** 2024-06-25

**Authors:** Amir Masoud Jafari-Nozad, Najmeh Jahani, Yoones Moniri

**Affiliations:** 1 Student Research Committee, Birjand University of Medical Sciences, Birjand, Iran; 2 Department of Gynecology, School of Medicine, Valiasr Hospital, Birjand University of Medical Sciences, Birjand, Iran; 3 Clinical Research Development Unit, Razi Hospital, Birjand University of Medical Sciences, Birjand, Iran

**Keywords:** Carcinoid tumor, Mature cystic teratoma, Malignant transformation, Neuroendocrine tumor, Ovarian neoplasm

## Abstract

**Background:**

Mature cystic teratomas (MCT) of the ovary are benign ovarian germ cell neoplasms. Malignant transformation is possible but rare and ovarian carcinoid tumors in MCT are among the most extremely rare subtypes.

**Case presentation:**

We report a case of a 60-year-old Iranian woman suffering from postmenopausal bleeding and hypogastric pain for the last 40 days. An adnexal mass was detected during the physical examination. Ultrasound imaging showed a (55 × 58) mm mass in the left ovary. Total abdominal hysterectomy, bilateral salpingooophorectomy and comprehensive staging surgery were performed for the patient. Intraoperative frozen section of the left ovarian mass was indicative of a malignant tumor. She was diagnosed with a carcinoid tumor with benign mucinous cystadenoma arising on MCT of the ovary, confirmed in the histopathology and immunohistochemistry examination. The tumor was classified as low grade and no chemotherapy cycles were considered. The patient was followed up long-term and no recurrence was observed during 14 months of examinations.

**Conclusion:**

Ovarian carcinoids arising from MCT are rare neuroendocrine neoplasms, and proper diagnosis of these tumors requires careful histopathology evaluation and appropriate examination. Therefore, it is necessary to consider these tumors as a possible differential diagnosis and evaluate them in individuals (especially postmenopausal women) who have abdominal pain or abnormal bleeding and a palpable mass.

## Introduction

Mature cystic teratomas of the ovary, MCTO for short, are benign ovarian neoplasms of well-differentiated germ cell layers [1, 2]. Malignant transformation is rare (only 1–3% of all MCTs), with most reported cases being squamous cell carcinoma (SCC) [3, 4]. Certain risk factors, such as tumor size, patient age, serum tumor markers level, and imaging results may increase the likelihood of malignant transformation [5]. Case reports have shown that malignant transformation of teratomas usually occurs in postmenopausal women, who are about 15 years older than women with conventional benign ovarian neoplasms [6]. Although most patients are asymptomatic, some may experience symptoms such as abnormal uterine bleeding, postmenopausal bleeding (PMB), abdominal pain, constipation, and a noticeable mass in the abdomen or pelvic area [7, 8].

Carcinoid tumors are slow-growing neoplasms originating from neuroendocrine cells. Ovarian carcinoid tumors and their malignant tumors in MCT are extremely rare [9, 10]. This study aimed to report a case of a mucinous cystadenoma and carcinoid tumor arising from an ovarian mature cystic teratoma in a 60-year-old woman, confirmed by postoperative histopathologic and immunohistochemistry (IHC) results.

## Case presentation

A 60-year-old postmenopausal Iranian woman, Para 6 Live 6, was presented to the obstetrics and gynecology department of Valiasr Hospital, Birjand, Iran, in early 2023 with PMB and hypogastric pain for the last 40 days. The pain was colicky in nature and non-positional. The patient described her vaginal bleeding as less intense than her prior menstrual cycle bleedings, with dark red color-clotted discharge. She did not complain about constipation, urinary frequency, decreased appetite, or weight loss. At the initial examination, her vital signs were within normal limits. The patient had no comorbidities, and there was no significant past medical history, except lumbar spinal stenosis, which was operated ten years ago. Her family history was unremarkable in terms of any cancers or gynecological problems. The patient said that she attained menopause 7 years back.

There was a palpable abdominopelvic mass in the left lower quadrant (LLQ) and hypogastric area on abdominal examination. We did an abdominal ultrasound, and the results were as follows: the uterus size was reported as (42** × **79) mm with thick (12 mm) and heterogeneous endometrium/a (58** × **55) mm lobulated mass with multiple cystic components and relatively thick septa was seen in the left ovary. Additional evaluation was carried out, involving laboratory tests and computed tomography (CT). Abdominal and pelvic contrast-enhanced CT confirmed a (6 × 5.5) cm mass in the left ovary, with no convincing metastases. Serum tumor markers (CEA, AFP and CA-125) were assessed and reported within normal limits. Regarding the imaging results and patient symptoms (abnormal virginal bleeding), we scheduled a dilatation and curettage (D&C). The pathologist reported the results as mixed endometrial-endocervical polyp with no malignancy.

Consent was obtained after informing the patient about the procedure, and the surgery was carried out as planned. Total abdominal hysterectomy (TAH), bilateral salpingo-oophorectomy (BSO), and comprehensive staging surgery were performed in the standard steps. During the surgery, a 6 × 5 cm multicystic tumor with solid growth and clear fluid was found, and an intraoperative frozen section specimen was sent to the histopathology laboratory. Post-operative recovery was uneventful, and the patient was discharged 1 week later.

**Fig. 1:**
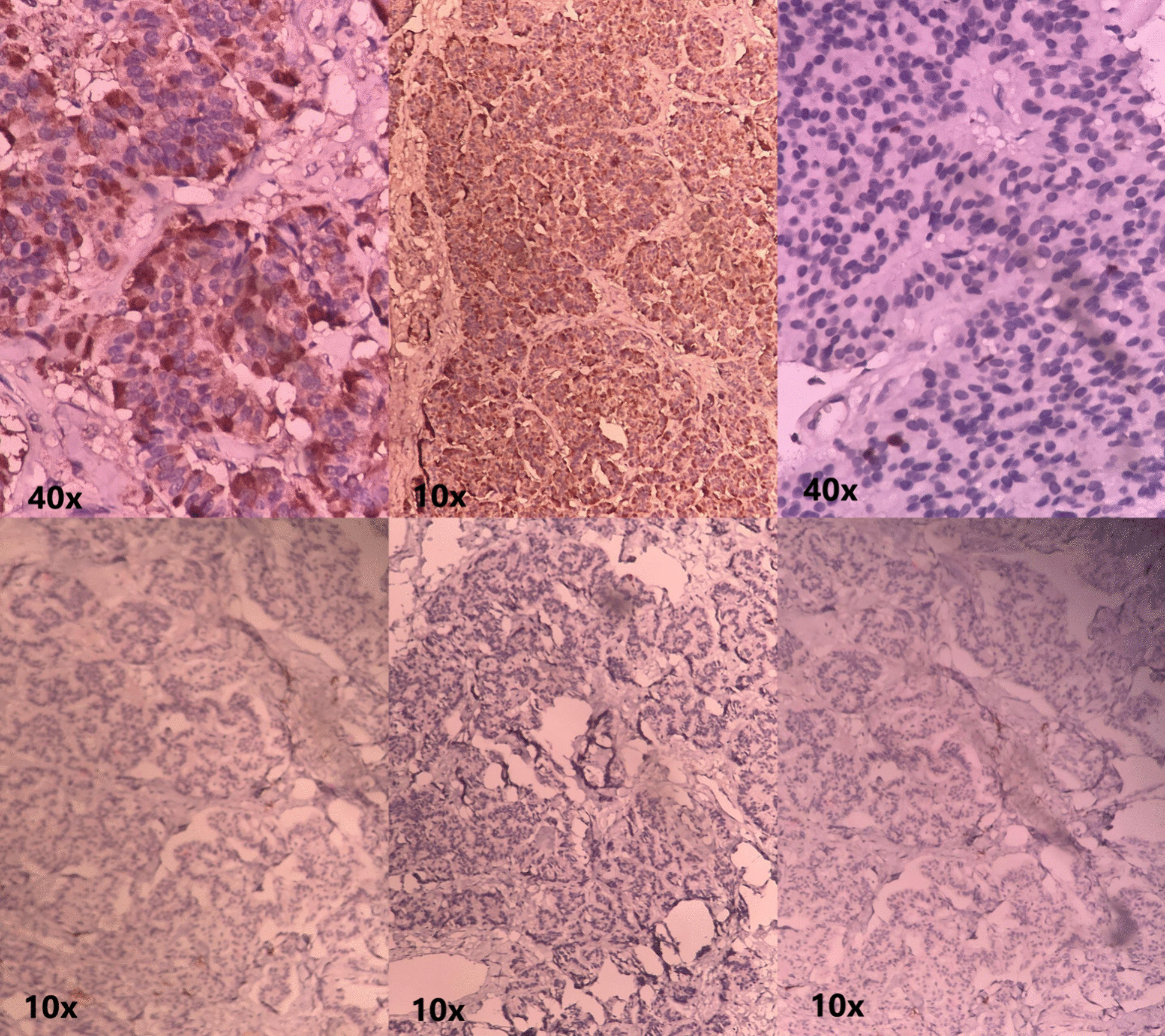
*Immunohistochemical Staining of the Ovarian Mass.* IHC results were indicative of a low grade carcinoid tumor with benign mucinous cystadenoma arising on MCT. Epithelial markers of the malignant cells: Positive for synaptophysin, chromogranin, and CK / Negative for CK20, CK7, inhibin, and PAX8 (the microscope magnification is labeled under each image, as 10 × or 40 ×)

The frozen section showed that the tumor was malignant, but it could not determine the exact pathological subtype. Based on the postoperative histopathological analysis and IHC findings, the tumor was classified as a low grade well-differentiated neuroendocrine tumor, grade 1 (carcinoid tumor) with benign mucinous cystadenoma (arising on MCT). The IHC report of epithelial markers indicated that the malignant cells were positive for synaptophysin, chromogranin, and CK. The cells were negative for CK20, CK7, inhibin, and PAX8. Chromogranin and synaptophysin are great discriminatory markers for carcinoid tumors. Both showed positivity in our case (Fig. 1: Immunohistochemical staining of the ovarian mass).

Regarding the teratoma being low grade, she received no further treatment, and we did not consider chemotherapy for the patient and sculled long-term follow-ups for any potential complications. Our follow-up plan consisted of a physical examination plus abdominal ultrasonography every 3 months. We also planned an abdominal and pelvic CT each 6 month. She experienced an uneventful clinical course with normal tumor markers and imaging during the follow-up. No evidence of recurrence was observed after 14 months of follow-up examinations.

## Discussion

Ovarian carcinoid tumors are rare low-grade neuroendocrine tumor, accounting for about 0.3% of all carcinoid tumors and less than 0.1% of all ovarian neoplasms [10, 11]. MCTO malignant transformation is possible but rare, with challenging diagnosis and management [12–14]. The growth of carcinoids within a teratoma is even rarer. These are tumors with low malignancy potential; however, their prognosis and clinical outcomes have not yet been determined [15]. Clinical symptoms are often non-specific, including abdominal pain, abnormal vaginal bleeding, and dysmenorrhea [16]. In the majority of cases, like our patient, primary ovarian carcinoid tumors are unilateral. However, it is worth mentioning that in 16%, the other ovary may display a cystic teratoma or a mucinous tumor.

Although malignant MCTO has a broad range of age distribution (14–79 years), prior literature indicated that most patients are postmenopausal women [17, 18]. Our patient was 60 years old. Tosuner et al. reported a case of a carcinoid tumor arising in MCTO in a 75‑year‑old woman suffering from groin pain [19]. In another report, Orsi and colleagues described a 65-year-old woman who was presented with an abdominal mass and raised CA-125 level [20]. Although most patients are postmenopausal and over 45 years old, there are some reports in the literature indicating that malignant transformation of MCTO can be seen in younger individuals. In 2016, Kim reported a carcinoid tumor arising from MCTO in a 25-year-old woman. She was admitted with a history of an asymptomatic pelvic mass four years ago that had increased in size recently. Ultrasonography demonstrated a hypoechoic cystic mass, and a CT scan revealed tumors in both ovaries (the right ovary: 7.0 × 4.5 cm/the left ovary: 16 × 14 cm) [10]. Another study demonstrated a 28-year-old female with ovarian carcinoid tumor who was treated with a conservative approach. The patient was asymptomatic and was referred for the routine annual gynecological checkups. Considering the age of the patient and to preserve her fertility, a laparoscopic approach was used to remove the ovarian mass, leaving the remaining ovary anatomy intact [21]. Depending on the patient's age, more aggressive surgical approaches, like TAH and BSO, may be considered as a reasonable option (mostly in postmenopausal women that have completed their childbearing) [22].

In younger individuals, the treatment options are even more challenging and nonspecific. Limited reports have been documented regarding carcinoid tumors developing in MCTO that were treated with conservative surgery. Interestingly, there was no evidence of disease recurrence after several months of follow-up. This suggests that a conservative strategy may be appropriate for young patients who wish to maintain their fertility [21]. In summary, the best and most effective approach for management of such malignant transformations is unclear, and there is no standard approach or direct comparison between available treatments. However, surgical removal is the initial step in almost all cases [18, 23].

## Conclusion

In conclusion, ovarian carcinoid tumors arising from MCTO are extremely rare. Preoperative diagnosis is challenging and most patients are diagnosed after pathological and IHC evaluations. Although the age distribution of these malignancies varies widely, most patients are postmenopausal women. Early detection and timely management are essential to improve patient outcomes. Given the high number of reported cases, it is strongly necessary for radiologists, pathologists, and clinicians to be aware of the possibility of MCTO malignant transformation.

## Data Availability

The data that support the findings of this study are available on request from the corresponding author.
